# Social isolation and loneliness and mortality among cancer survivors: a prospective cohort study

**DOI:** 10.3389/fonc.2026.1908858

**Published:** 2026-09-09

**Authors:** Ran Ran, Manyun Tang, Ruohan Liu, Jin Yang, Yingying Liu

**Affiliations:** 1 Department of Cardiovascular Surgery, The First Affiliated Hospital of Xi’an Jiaotong University, Xi’an, China; 2 The Comprehensive Breast Care Center, The Second Affiliated Hospital of Xi’an Jiaotong University, Xi’an, China; 3 Cancer Center, The First Affiliated Hospital of Xi’an Jiaotong University, Xi’an, China; 4 Department of Cardiovascular Medicine, The First Affiliated Hospital of Xi’an Jiaotong University, Xi’an, China

**Keywords:** cancer survivors, social isolation, loneliness, social connection, mortality

## Abstract

**Background:**

Social isolation and loneliness are associated with mortality, but their impact on cause-specific mortality in cancer survivors remains unclear.

**Methods:**

This cohort study enrolled UK Biobank participants with cancer at baseline, followed up from March 2006 to September 2024. Social isolation and loneliness were assessed by self-reported questionnaires. Outcomes included all-cause, cancer-specific, and cardiovascular disease (CVD)-related mortality. Associations were evaluated using Cox proportional hazards models.

**Results:**

A total of 32,524 participants were included in this study (60.2% women; mean age, 60.3 years). During a median 15.7-year follow-up, 5,945 deaths occurred. Social isolation was significantly associated with increased all-cause (HR, 1.22; 95%CI, 1.12–1.32), cancer-specific (HR, 1.16; 95%CI, 1.04–1.28), and CVD-related (HR, 1.32; 95%CI, 1.07–1.63) mortality. Loneliness was not associated with all-cause mortality (HR, 0.98; 95%CI, 0.93–1.04), but independently associated with increased CVD-related mortality (HR, 1.26; 95%CI, 1.08–1.47). Exploratory analyses identified distinct prognostic impacts of specific isolation measures: living alone was linked to higher all-cause and non-cancer mortality across most cancer types, while weekly social activities and monthly family/friends visits were protective.

**Conclusions:**

Social isolation was associated with increased risks of all-cause, cancer-specific, and CVD-related mortality. Loneliness was associated with elevated CVD-related mortality.

## 1 Introduction

With advancements in early detection and treatment, coupled with an aging population, the population of cancer survivors is growing. In the United States alone, an estimated 18.6 million cancer survivors (5% of the population) are projected by 2025 (1). Compared to the general population, cancer survivors face elevated mortality risks from recurrence, cardiovascular diseases (CVDs), and treatment-related complications (2, 3).

Mounting evidence suggests that social isolation and loneliness are critical determinants of mortality in the general population, comparable to conventional factors such as hypertension, smoking, and obesity (4–8). Social isolation denotes objectively limited social networks and interactions, while loneliness reflects subjective dissatisfaction with relationship quality or unmet social needs. Both are associated with adverse health outcomes, including CVDs, mental disorders, and all-cause mortality (7, 9–12). A 2015 meta-analysis showed that social isolation and loneliness increased all-cause mortality by 26% and 29%, respectively (7). Another meta-analysis demonstrated that adverse social relationships increased the risk of coronary heart disease by 29% and stroke by 32% (9). However, the specific impact of social isolation and loneliness on cancer survivorship remains less clear, with existing studies reporting inconsistent findings (13–16). Among cancer survivors, robust social supports may facilitate treatment adherence, emotional resilience, and improved quality of life, whereas isolation exacerbates comorbidities and mental health decline (17–20). Despite these insights, serious limitations persist. First, most studies focus on the general population and neglect the specificity of cancer survivors. Second, the correlation between social isolation and loneliness is weak, yet previous analyses often conflate these two concepts, obscuring their distinct effects. Third, large-scale prospective studies on the differential impacts of social isolation and loneliness on cause-specific mortality are scarce.

To address these gaps, we conducted a prospective population-based cohort study utilizing UK Biobank data from cancer survivors. This study aimed to (1) quantify the associations of social isolation and loneliness with all-cause, cancer-specific, CVD-related, and other-cause mortality among cancer survivors; (2) examine these associations across specific cancer types; and (3) provide evidence-based recommendations for enhancing psychosocial support system to improve the long-term survival outcomes in cancer survivors.

## 2 Materials and methods

### 2.1 Study design and participants

The UK Biobank is a large-scale prospective population-based cohort study involving over 500,000 participants aged 40–70 years who were enrolled between 2006 and 2010 (21). For individuals with a history of cancer, diagnostic information and dates were obtained from the National Cancer Registries. The diagnosis was determined using the 9th revision of the International Classification of Diseases (ICD-9) codes (140–208) and/or ICD-10 codes (C00–C97). The analysis excluded participants without a cancer diagnosis at baseline (n = 467,249) and those lacking complete data on social isolation and loneliness (n = 2,382) (**Supplementary Figure 1**). Ethical approval for the study was granted by the North West Multicenter Research Ethics Committee (approval no. 16/NW/0274), and all participants provided written informed consent prior to enrollment (21).

### 2.2 Definition of social isolation and loneliness scales

The social isolation index was derived from three items on the UK Biobank touchscreen questionnaire: (1) “Including yourself, how many people are living together in your household?” (1 point for living alone); (2) “How often do you visit friends or family or have them visit you?” (1 point for friends or family visit less than once a month); and (3) “Which of the following [leisure/social activities] do you attend once a week or more often?” (1 point for no weekly participation in social activities). The total score ranged from 0 to 3. Participants with a score of 2 or 3 were classified as having high social isolation, whereas those with a score of 0 or 1 were classified as having low social isolation.

The loneliness index was assessed using two items: “Do you often feel lonely?” (1 point for yes) and “How often are you able to confide in someone close to you?” (1 point for confiding less than once a month). Participants with a score of 2 were defined as having high loneliness, and those with a score of 0 or 1 as having low loneliness. Scoring methods for social isolation and loneliness are detailed in **Supplementary Table 1**. Similar scales have been applied previous UK Biobank studies (22).

### 2.3 Outcomes assessment and follow-up

The primary outcomes of this study included all-cause, cancer-specific, and CVD-related mortality. The end date for follow-up was determined as the date of the baseline to death or the censoring date (September 30, 2024), whichever occurred first. The results were categorized according to the International Statistical Classification of Diseases and Related Health Problems, Tenth Revision (ICD-10). We analyzed mortality for all-cause, cancer-specific (codes C00 to C97), CVD-related (codes I00 to I99), and other-cause (all remaining ICD-10 codes).

### 2.4 Covariates

Baseline self-reported age, sex (female, male), ethnicity (white, non-white), income levels (Level 1: <18000; Level 2: 18000–30999; Level 3: 31000–51999; Level 4: >52000 £/year); employment status (paid employment/self-employed; retired or unemployed); education attainment (college degree or above; high school or equivalent; less than high school); smoking status (never, previous, current); alcohol intake [low (<3 times/week), high (≥3 times/week)] (23); sleep duration (0–6, 7–8, >8 hours); International Physical Activity Questionnaire (IPAQ) physical activity (low, moderate, and high) and Townsend deprivation index (TDI), body mass index (BMI) were collected directly from UK Biobank. The healthy diet score was calculated based on previous studies (24, 25), with ≥3 classified as healthy diet and <3 as unhealthy diet (**Supplementary Table 2**). Chronic diseases (hypertension, hyperlipidemia, diabetes, and CVD) were defined as having a self-reported history or being diagnosed by ICD-10 codes (26). Depressive symptoms were evaluated using the frequency of four items from the Patient Health Questionnaire (27): (1) depressed mood, (2) disinterest or absence of enthusiasm, (3) tenseness or restlessness, and (4) tiredness or lethargy over the past two weeks. Self-rated health was measured through the question “In general how would you rate your overall health?” with responses scored on a 4-point scale (1=poor to 4=excellent). Further details of these measures are available via the UK Biobank website and **Supplementary Table 3** (28).

### 2.5 Statistical analysis

Continuous variables were presented as mean and standard deviation (SD) and categorical variables were presented as counts (percentages). Baseline characteristics were compared between low and high social isolation/loneliness groups using χ² or Wilcoxon rank-sum tests. Cox proportional hazards models were employed to examine the association of social isolation and loneliness with risk of all-cause and cause-specific mortality, with follow-up time from enrollment to event occurrence or the censoring date as the timescale. The validity of the proportional hazards assumption was evaluated using Schoenfeld residuals and Kaplan-Meier methods, and all examinations met the prespecified criteria. We constructed five Cox models. Model 1 was adjusted for demographic factors (age, sex, and ethnicity). Model 2 was further adjusted for socioeconomic factors (income, employment, education, and TDI). In Model 3, we further added lifestyle factors (smoking status, alcohol intake, diet, sleep duration, IPAQ, BMI). Model 4 was further adjusted for health status (depressive symptoms [hypertension, diabetes, hyperlipidemia, CVD and chronic lung disease, depressed mood, unenthusiasm/disinterest, tenseness/restlessness, tiredness/lethargy], self-rated health, and time since cancer diagnosis). Model 5 was fully adjusted to include loneliness or social isolation. Missing data were handled using multiple imputation by chained equations (29, 30). The number and percentage of missing data for major covariate before multiple imputation are shown in **Supplementary Table 4**. The GVIF values (range: 1.02–1.95) were substantially lower than the multicollinearity threshold of 10 (**Supplementary Table 5**), supporting the inclusion of all social connection measures as independent variables in the models. **Supplementary Figure 2** presents a directed acyclic graph explaining the associations among the exposures, the outcome, and the covariates. In addition, the Kaplan-Meier method was used to evaluate cumulative all-cause and cause-specific mortality by levels of social isolation/loneliness, with between-group differences evaluated using Log-rank tests.

We investigated the interaction between social isolation and loneliness on mortality through multiplicative and additive scales. Multiplicative interaction was conducted using cross terms, while the additive interaction analysis was performed by calculating the relative excess risk due to interaction (RERI), attribution proportion due to interaction (AP) and synergy index (SI) (30, 31). We also conducted stratified analyses based on social isolation or loneliness status. Participants with low social isolation and low loneliness were used as the reference group in the joint analysis.

To examine potential variations in these association across different subgroups, we conducted subgroup analyses by age (<60 or ≥60 years old), sex (women or men), ethnicity (white or non-white), BMI (<25.0, 25.0–29.9, or ≥30.0 kg/m^2^), TDI (tertiles), chronic diseases at baseline (yes or no), time since cancer diagnosis (>5 years before baseline or ≤5 years prior to baseline).

Four sensitivity analyses were performed to assess the robustness of the findings: 1) limiting analyses to participants with complete covariate data to evaluate potential biases from missing information; 2) excluding participants who died within 2 years after recruitment to mitigate potential reverse causality; 3) social isolation (score of 0–3) and loneliness (0–2) were analyzed as continuous variables included in Cox models; 4) employing Fine-Gray subdistribution hazard models, considering non-cancer mortality as a cancer mortality competing events.

All statistical analyses were performed using R software (version 4.2.3). A two-sided P value of less than 0.05 was considered statistically significant.

## 3 Results

### 3.1 Baseline characteristics of participants

Of the 32,524 cancer survivors (mean [SD] age, 60.3 [6.9] years; 19,583 [60.2%] women; 31,024 [95.4%] white participants), 2,974 participants (9.1%) had high social isolation, and 10001 (30.7%) had high loneliness. Cancer survivors with high levels of either social isolation or loneliness exhibited similar adverse socioeconomic and poor health profiles compared to their low counterparts (**Table 1**). Both groups showed significantly higher proportions of non-white ethnicity, higher educational attainment but lower incomes, higher TDI, and more unhealthy lifestyles (smoking, unhealthy diet, shorter sleep duration, and lower levels of physical activity), as well as higher BMI and higher comorbidity burden, including hypertension, diabetes, hyperlipidemia, CVD, and chronic lung disease. Moreover, retirement and unemployment rates were higher in the high loneliness group compared to the low loneliness group.

**Table 1:** Baseline characteristics of cancer survivors by social isolation and loneliness.

Characteristics	Social isolation		*P* value	Loneliness		*P* value	Total (n=32524) (%)
	Low (n = 29550) (%)	High (n = 2974) (%)		Low (n = 22523) (%)	High (n = 10001) (%)		
Sex							
Male	11773 (39.8)	1168 (39.3)	0.560	8970 (39.8)	3971 (39.7)	0.838	12941 (39.8)
Female	17777 (60.2)	1806 (60.7)		13553 (60.2)	6030 (60.3)		19583 (60.2)
Age, years	60.3 (6.9)	60.2 (6.6)	0.164	60.3 (6.9)	60.3 (6.9)	0.924	60.3 (6.9)
Ethnicity							
White	28249 (95.6)	2775 (93.3)	<0.001	21573 (95.8)	9451 (94.5)	<0.001	31024 (95.4)
Non-White	1301 (4.4)	199 (6.7)		950 (4.2)	550 (5.5)		1500 (4.6)
Income ^a^							
Level1	8289 (28.1)	1516 (51.0)	<0.001	5925 (26.3)	3880 (38.8)	<0.001	9805 (30.1)
Level2	8698 (29.4)	708 (23.8)		6571 (29.2)	2835 (28.3)		9406 (28.9)
Level3	7012 (23.7)	461 (15.5)		5472 (24.3)	2001 (20.0)		7473 (23.0)
Level4	5551 (18.8)	289 (9.7)		4555 (20.2)	1285 (12.8)		5840 (18.0)
Employment							
Paid employment/Self-employed	12090 (40.9)	1234 (41.5)	0.553	9417 (41.8)	3907 (39.1)	<0.001	13324 (41.0)
Retired or unemployed	17460 (59.1)	1740 (58.5)		13106 (58.2)	6094 (60.9)		19200 (59.0)
Education							
College degree or above	5999 (20.3)	852 (28.6)	<0.001	4275 (19.0)	2576 (25.8)	<0.001	6851 (21.1)
High school or equivalent	14432 (48.8)	1313 (44.1)		10857 (48.2)	4888 (48.9)		15745 (48.4)
Less than high school	9119 (30.9)	809 (27.2)		7391 (32.8)	2537 (25.4)		9928 (30.5)
Townsend deprivation index ^b^	-1.7 (2.8)	-0.2 (3.4)	<0.001	-1.8 (2.8)	-1.2 (3.1)	<0.001	-1.6 (2.9)
BMI, kg/m^2^	27.3 (4.7)	27.6 (5.3)	0.001	27.2 (4.6)	27.7 (5.0)	<0.001	27.3 (4.7)
Smoking status							
Never	15545 (52.6)	1363 (45.8)	<0.001	12009 (53.3)	4899 (49.0)	<0.001	16908 (52.0)
Previous	11680 (39.5)	1109 (37.3)		8862 (39.3)	3927 (39.3)		12789 (39.3)
Current	2325 (7.9)	502 (16.9)		1652 (7.3)	1175 (11.7)		2827 (8.7)
Alcohol intake							
Low	16342 (55.3)	2044 (68.7)	<0.001	12296 (54.6)	6090 (60.9)	<0.001	18386 (56.5)
High ^c^	13208 (44.7)	930 (31.3)		10227 (45.4)	3911 (39.1)		14138 (43.5)
Healthy diet							
Less healthy	7890 (26.7)	915 (30.8)	<0.001	5775 (25.6)	3030 (30.3)	<0.001	8805 (27.1)
Healthy	21660 (73.3)	2059 (69.2)		16748 (74.4)	6971 (69.7)		23719 (72.9)
Sleep duration, hours							
0–6	6739 (22.8)	915 (30.8)	<0.001	4699 (20.9)	2955 (29.5)	<0.001	7654 (23.5)
7–8	19853 (67.2)	1740 (58.5)		15582 (69.2)	6011 (60.1)		21593 (66.4)
>8	2958 (10.0)	319 (10.7)		2242 (10.0)	1035 (10.3)		3277 (10.1)
IPAQ							
Low	5419 (18.3)	766 (25.8)	<0.001	4152 (18.4)	2157 (21.6)	<0.001	13509 (41.5)
Moderate	12283 (41.6)	1255 (42.2)		9370 (41.6)	4139 (41.4)		12706 (39.1)
High	11848 (40.1)	953 (32.0)		9001 (40.0)	3705 (37.0)		27.3 (4.7)
Hypertension	9296 (31.5)	1063 (35.7)	<0.001	6969 (30.9)	3390 (33.9)	<0.001	10359 (31.9)
Diabetes	1627 (5.5)	240 (8.1)	<0.001	1157 (5.1)	710 (7.1)	<0.001	1867 (5.7)
Hyperlipidemia	6013 (20.3)	709 (23.8)	<0.001	4482 (19.9)	2240 (22.4)	<0.001	6722 (20.7)
CVD	1890 (6.4)	2974 (9.1)	<0.001	1325 (5.9)	832 (8.3)	<0.001	2160 (6.6)
Chronic lung disease	1887 (6.4)	270 (9.1)	<0.001	653 (2.9)	479 (4.8)	<0.001	1132 (3.5)
Self-rated health	2.7 (0.7)	2.5 (0.8)	<0.001	2.8 (0.7)	2.6 (0.8)	<0.001	2.7 (0.8)
Depressed mood	1.3 (0.6)	1.5 (0.8)	<0.001	1.2 (0.4)	1.5 (0.8)	<0.001	1.3 (0.6)
Unenthusiasm/disinterest	1.2 (0.6)	1.5 (0.8)	<0.001	1.2 (0.5)	1.5 (0.7)	<0.001	1.3 (0.6)
Tenseness/restlessness	1.3 (0.6)	1.4 (0.7)	<0.001	1.2 (0.5)	1.5 (0.7)	<0.001	1.3 (0.6)
Tiredness/lethargy	1.7 (0.8)	2.0 (1.0)	<0.001	1.6 (0.8)	2.0 (1.0)	<0.001	1.7 (0.9)
Time since diagnosis, months	94.8 (85.9)	97.3 (88.8)	0.134	93.9 (85.8)	97.6 (87.0)	<0.001	95.1 (86.2)

Figures given are N (column %) or mean (SD).

Sex, ethnicity, income, employment, education, smoking status, alcohol intake, health diet, sleep duration and chronic disease were treated as categorical variables and the other risk factors as continuous variables in the analyses. Depressed mood, unenthusiasm/disinterest, tenseness/restlessness, tiredness/lethargy, self-rated health all ranged from 1 to 4.

BMI, body mass index; CVD, cardiovascular disease

^a^ Annual income is divided into four levels: Level1, less than £18000; Level2, £18000 to £30999; Level3, £31000 to £51999; Level4, greater than £52000

^b^ A composite area-level socioeconomic deprivation measure derived from four standardized UK census indicators—unemployment rate (percentage of working-age adults unemployed), non-car ownership (percentage of households without a car), non-home ownership (percentage of households renting), and household overcrowding (percentage of households exceeding one person per room). These components are summed to produce a continuous score where higher positive values indicate greater material deprivation, while negative values reflect relative affluence.

^c^ High alcohol intake is defined as drinking three or more times per week.

### 3.2 Social isolation and loneliness with the risk of mortality among all cancer survivors

After an overall median follow-up time of 15.7 years (IQR: 14.4–16.2 years), 5,945 (18.3%) all-cause deaths (50.6% male) were recorded, of which 3,965 (66.7%) were due to cancer and 762 (12.8%) to CVD.

**Figure 1:**
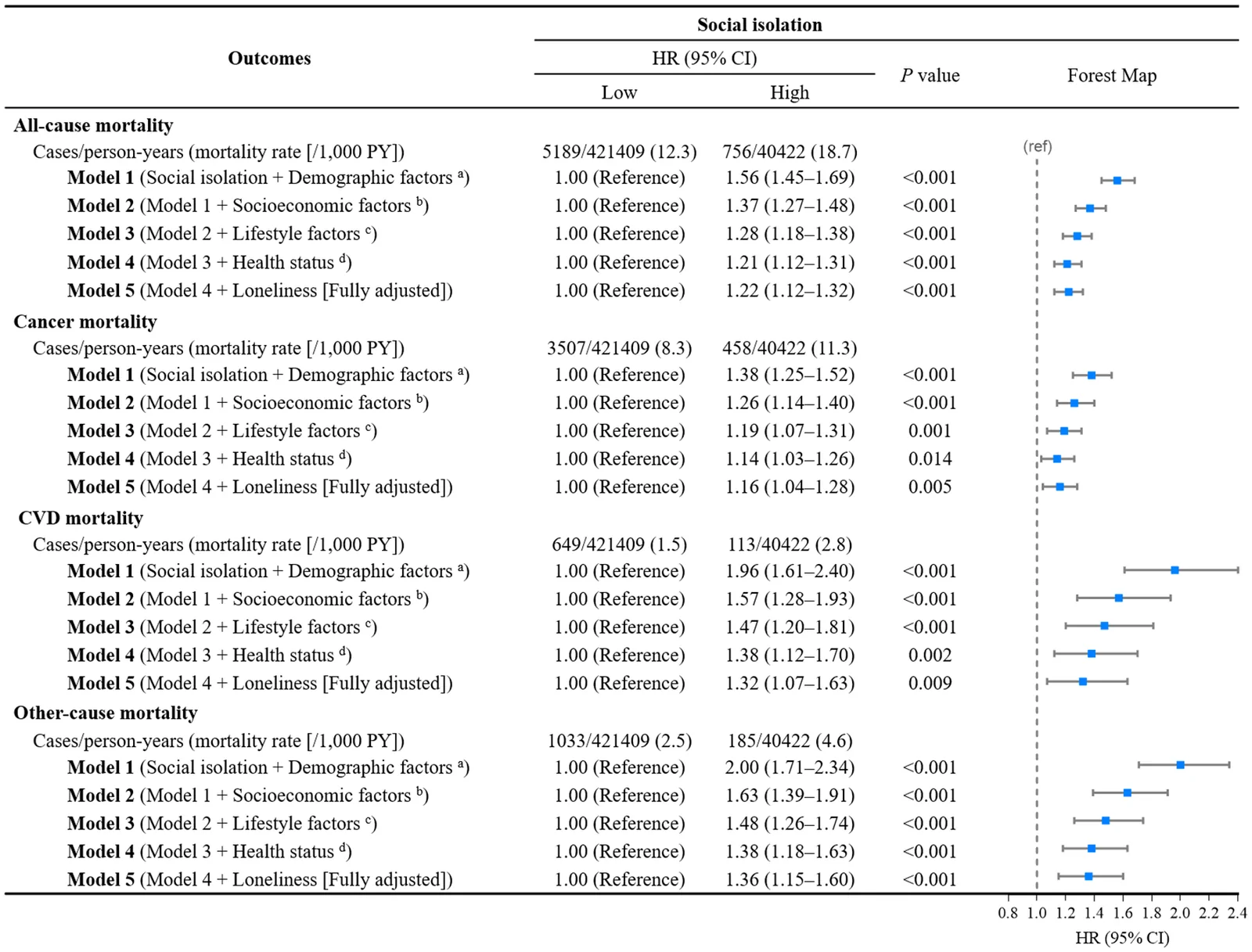
Models of association between social isolation and mortality among cancer survivors. ^a^Demographic factors: age, sex, ethnicity. ^b^Socioeconomic factors: income, employment, education, Townsend deprivation index. ^c^Lifestyle factors: smoking status, alcohol intake, health diet, sleep duration, IPAQ, BMI. ^d^Health status: hypertension, diabetes, hyperlipidemia, CVD, chronic lung disease, depressive symptoms (depressed mood, unenthusiasm/disinterest, tenseness/restlessness, tiredness/lethargy), self-rated health, time since cancer diagnosis. HR, hazard ratio; CI, confidence interval; IPAQ, International Physical Activity Questionnaire; ref, reference; PY, person-years.

In Cox models with minimally adjusted (Model 1) to fully adjusted (Model 5), high social isolation remained significantly associated with higher all-cause mortality (**Figure 1**). The attenuation of HR (from 1.56 to 1.21) across models indicated that socioeconomic, lifestyle, and health factors partially explained this association. Similar trends were observed for cancer-specific, CVD-related and other-cause mortality. For loneliness, the high loneliness group was significantly associated with increased all-cause mortality in Model 1 (HR, 1.20; 95%CI, 1.14–1.27) and Model 2 (HR, 1.10; 1.04–1.16) (**Supplementary Figure 3**). After sequential inclusion of lifestyle factors and health status in the model, the HRs reduced to non-significant, indicating that social disadvantage and comorbid health problems may largely account for this association. Loneliness was consistently associated with an elevated risk of CVD-related mortality across all models, albeit with decreased HRs. In contrast, its association with cancer-specific mortality shifted from null to protective after full adjustment. The association between social isolation and mortality risk remained stable across different levels of loneliness, whereas the effects of loneliness on cancer-specific and CVD-related mortality were observed only in the low social isolation group (**Supplementary Tables 6, 7**). Cancer survivors with both high levels of social isolation and loneliness have a 65% increase in CVD-related mortality compared to those with low levels of both. Social isolation was associated with a higher risk of all-cause mortality, regardless of loneliness status (**Supplementary Table 8**). Furthermore, there was a lack of evidence for an interaction between social isolation and loneliness for both all-cause and cause-specific mortality (**Supplementary Table 8**). The cumulative curves illustrated the cumulative hazard of all-cause and cause-specific mortality among cancer survivors by social isolation and loneliness (**Supplementary Figures 4**, **5**). Moreover, almost all components of social isolation showed significant correlations with all-cause and cause-specific mortality (**Supplementary Table 9**).

### 3.3 Social isolation and loneliness with the risk of mortality among specific cancer survivors

**Figure 2:**
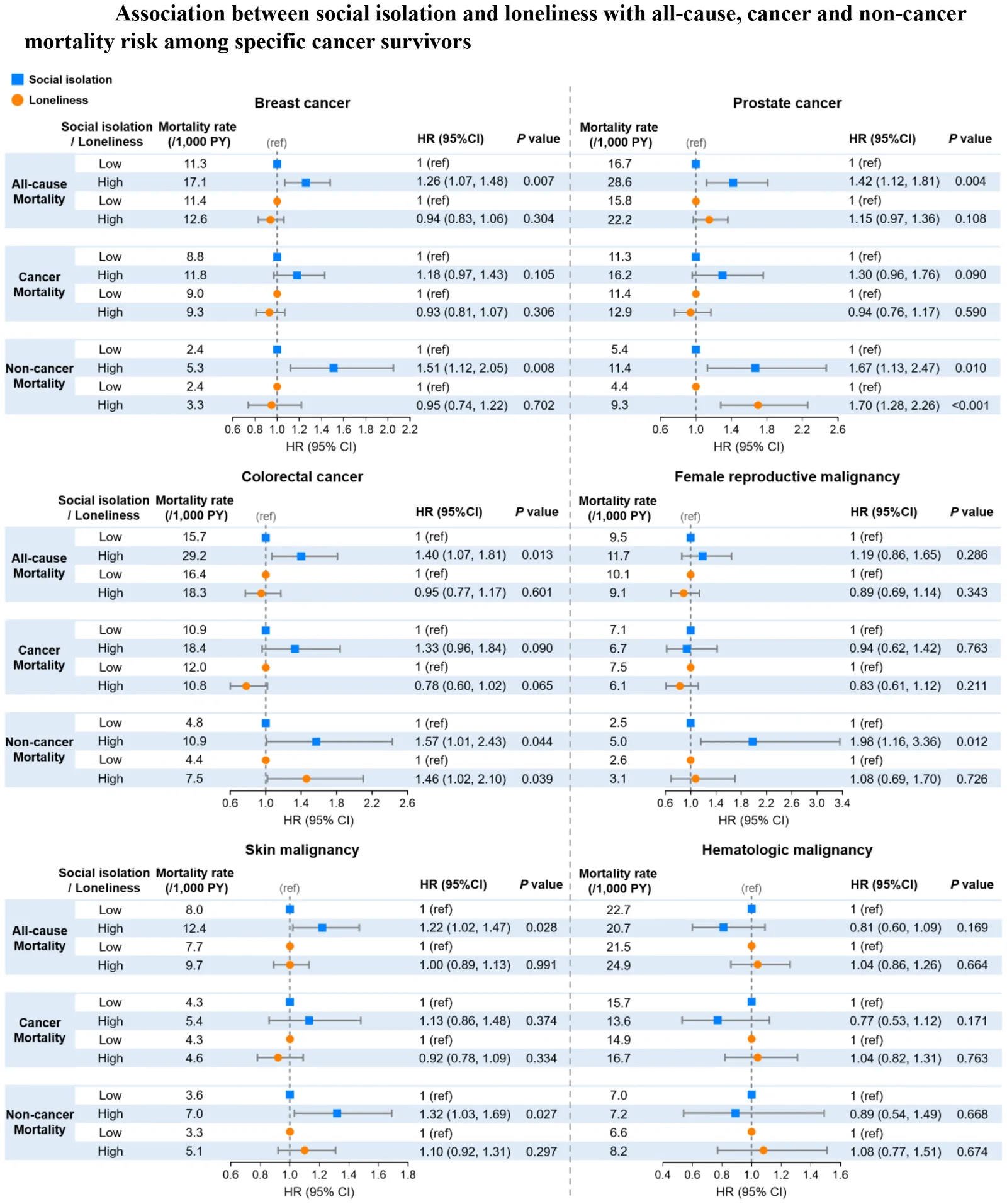
Association between social isolation and loneliness with all-cause, cancer and non-cancer mortality risk among specific cancer survivors. HR, hazard ratio; CI, confidence interval; ref, reference; PY, person-years. Multivariable model: adjusted for age, sex, and ethnicity, income, employment, education, Townsend deprivation index, smoking status, alcohol intake, diet, sleep duration, IPAQ, BMI, hypertension, diabetes, hyperlipidemia, CVD, chronic lung disease, depressed mood, unenthusiasm/disinterest, tenseness/restlessness, tiredness/lethargy, self-rated health, and time since cancer diagnosis.

Stratified analyses by primary tumor site revealed significant associations between social isolation and mortality outcomes (**Figure 2**). Among 8,340 breast cancer survivors, high social isolation predicted increased all-cause mortality (HR, 1.26, 95%CI, 1.07–1.48), with similar patterns observed in prostate (n=2,841; HR, 1.42; 1.12–1.81), colorectal (n=2,034; HR, 1.40; 1.07–1.81), and skin cancer survivors (n=10,741; HR, 1.22; 1.02–1.47). Due to the limited number of CVD-related and other-cause deaths in specific cancers, these outcomes were combined into a composite non-cancer mortality endpoint for analysis. High social isolation was associated with elevated non-cancer mortality across multiple cancers: breast (HR, 1.51; 1.12–2.05), prostate (HR, 1.67; 1.13–2.47), colorectal (HR, 1.57; 1.01–2.43), female reproductive malignancies (n=2,352; HR, 1.98; 1.16–3.36), and skin cancers (HR, 1.32; 1.03–1.69). However, social isolation showed no significant association with cancer-specific mortality for any tumor type. Additionally, Loneliness specifically predicted higher non-cancer mortality in prostate (HR, 1.70, 1.28–2.26) and colorectal cancer survivors (HR, 1.46, 1.02–2.10).

### 3.4 *Post hoc* analysis of the components of social isolation among specific cancer survivors

*Post hoc*, exploratory analysis examined the association of three components of social isolation (frequency of friends/family visits, weekly social activities, and living alone) and mortality among breast, prostate, colorectal, and skin malignancy survivors. In fully adjusted models, living alone independently predicted increased all-cause (prostate: HR, 1.30; 1.06–1.59; colorectal: HR, 1.35; 1.07–1.71; and skin: HR, 1.21; 1.06–1.40) and non-cancer mortality (breast: HR, 1.36; 95%CI, 1.06–1.75; prostate: HR, 1.51; 1.07–2.12; colorectal: HR, 1.84; 1.26–2.71; and skin: HR, 1.29; 1.06–1.57) across most cancer types. Weekly social activities were protective against all-cause (breast: HR, 0.87; 0.77–0.97; colorectal: HR, 0.75; 0.62–0.91) and cancer-specific mortality (breast: HR, 0.86; 0.75–0.98; colorectal: HR, 0.76; 0.60–0.96). Furthermore, monthly friends/family visits were associated with reduced all-cause (breast: HR, 0.75; 0.61–0.92) and non-cancer mortality (breast: HR, 0.55; 0.36–0.82; prostate: HR, 0.61; 0.41–0.89; skin: HR, 0.71; 0.54–0.94) (**Supplementary Figures 6**–**S9**).

In addition, the baseline prevalence of social isolation and loneliness across different cancer subtypes was also assessed (**Supplementary Table 10**). Overall, the prevalence of high loneliness was substantially higher than that of high social isolation across all cancer types. Notable variations in the prevalence of high social isolation and loneliness were observed across cancer subtypes. Specifically, patients with female reproductive malignancy had the highest prevalence of both social isolation (11.9%) and loneliness (33.9%) among all cancer types, whereas patients with skin malignancy had the lowest prevalence of social isolation (7.6%), and those with prostate cancer had the lowest prevalence of loneliness (29.7%).

### 3.5 Subgroup analyses

**Figure 3:**
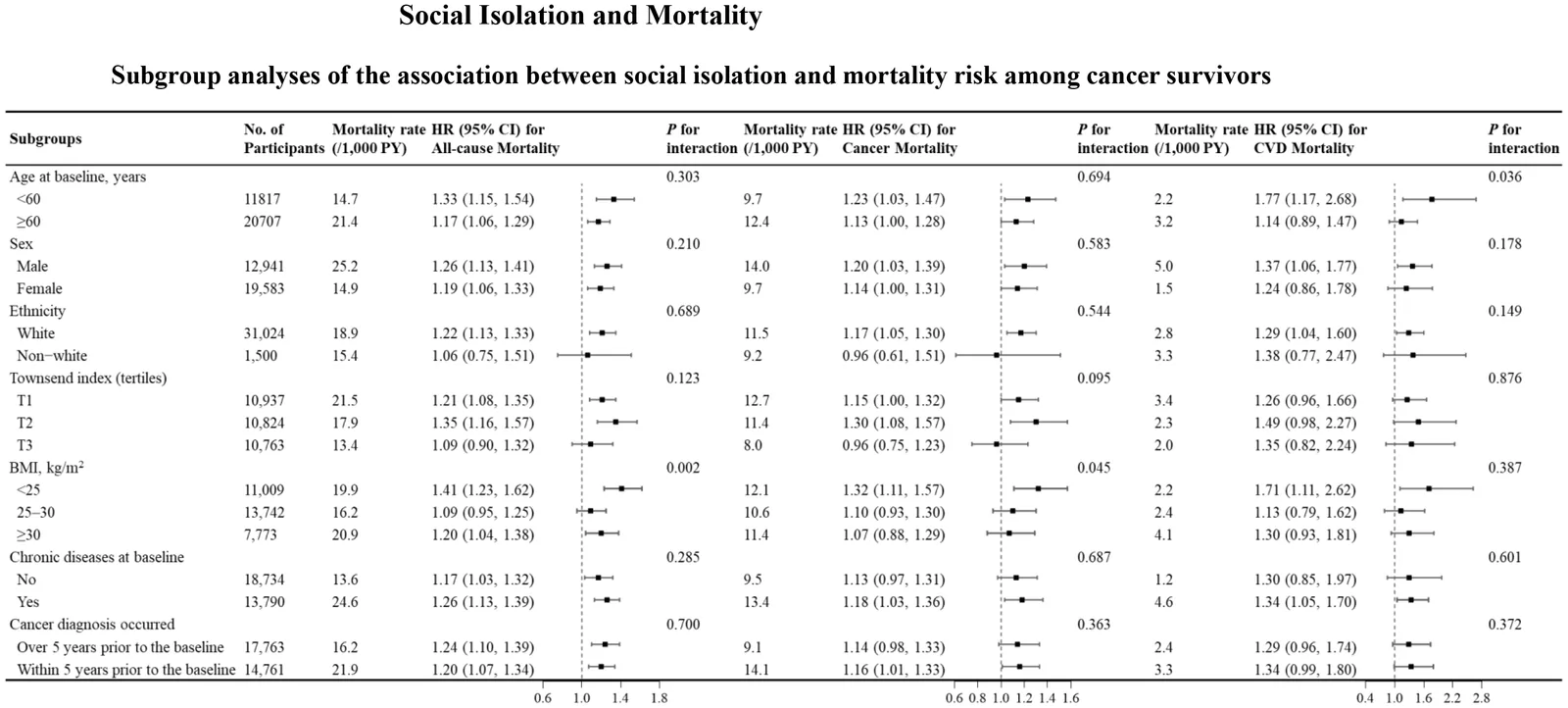
Subgroup analyses of the association between social isolation and mortality risk among cancer survivors. The dots represent the HRs for high versus low social isolation within the subgroups, and the horizontal lines indicate the 95% CIs. BMI, body mass index; CVD, cardiovascular disease; HR, hazard ratio; CI, confidence interval; ref, reference; PY, person-years.

As presented in **Supplementary Table 11**, the prevalence of high social isolation and high loneliness was higher among non-white individuals, those with socioeconomic disadvantage, individuals with obesity, and those with chronic diseases at baseline. Consistent associations of social isolation and loneliness with mortality were observed for subgroup analyses (**Figure 3**; **Supplementary Figure 10**). High social isolation showed a stronger association with CVD mortality in younger (<60 years) versus older (≥60 years) participants (*P* for interaction = 0.036). High social isolation exhibited more pronounced all-cause and cancer mortality risks in normal/lower BMI participants (*P* for interaction = 0.002 and 0.045, respectively). Furthermore, the impact of high loneliness on all-cause mortality was more prominent among participants with older age (≥60 years) and those with chronic disease at baseline (*P* for interaction = 0.030 and 0.045, respectively). High loneliness showed a stronger association with CVD mortality in male versus female participants (*P* for interaction=0.005).

### 3.6 Sensitivity analyses

The associations of social isolation and loneliness with mortality risk did not change significantly when we excluded participants with missing covariate data (**Supplementary Tables 12, 13**). After excluding participants who died within two years of recruitment, the results remained concordant with the main analysis (**Supplementary Tables 14, 15**). Moreover, social isolation and loneliness were included as continuous variables in fully adjusted Cox models, and the results remained robust (**Supplementary Table 16**). Fine-Gray competing risks analyses corroborated the main findings: the high social isolation group had significantly higher cumulative hazard of cancer mortality than the low isolation group, while no significant difference was observed between loneliness groups (**Supplementary Figure 11**).

## 4 Discussion

Our study explored the association of social isolation and loneliness with survival outcomes among cancer survivors in the UK Biobank. The findings demonstrated that social isolation was significantly associated with increased risks of all-cause, cancer-specific, and CVD-related mortality among overall cancer survivors, which were independent of baseline risk factors such as demographics, socioeconomics, lifestyles, comorbidities, and depressive symptoms. However, among specific cancer survivors, social isolation was significantly associated with all-cause and non-cancer mortality, and not with cancer mortality. Loneliness was associated with all-cause mortality in minimally adjusted models; this association reduced to non-significance after full adjustment for baseline risk factors. However, loneliness was independently associated with increased CVD mortality but negatively related to cancer mortality. Further exploratory analysis suggested that components of social isolation— living alone, weekly social activities, and monthly family/friends visits, all had independent prognostic significance, albeit with different roles in several high-incidence solid malignancies.

To the best of our knowledge, this is the first study specifically investigating the associations of social isolation and loneliness with all-cause and cause-specific mortality in cancer survivors. Our study indicated that social isolation was more strongly associated with mortality than loneliness, supported by several previous studies in the general population (22, 31). A large cohort study from the UK Biobank showed that social isolation was correlated with a 26% increased risk of all-cause mortality in the general population after full adjustment. However, the association between loneliness and mortality was entirely attributable to risk factors, with a fully adjusted HR of 0.99 (22). Another UK longitudinal study found that social isolation remained significantly associated with mortality (HR, 1.26; 95% CI, 1.08–1.48) after adjusting for demographic factors and baseline health, whereas loneliness did not (31).

We observed that social isolation and loneliness were more strongly associated with elevated risks of CVD-related or non-cancer mortality than with cancer-specific mortality in cancer survivors. These findings are supported by a prospective cohort of cancer survivors, which identified social determinants of health (SDOH) as independent predictors of all-cause, cancer, and cardiovascular mortality. Notably, worse social cohesion (one of the components of SDOH) correlated with all-cause and cardiovascular mortality, but not with cancer mortality (14). In addition, several studies have reported an association of social isolation and loneliness with increased incident CVD (9, 10, 32–35). Our study provides the first evidence that social isolation and loneliness significantly associated with a 32% and 26% higher risk of CVD mortality in cancer survivors, respectively, with particularly pronounced effects among younger individuals and males. This is concordant with previous evidence on the association between social vulnerability and mortality caused by comorbid cancer and CVD (36). A study from nationally representative US data suggested that unfavorable SDOH profiles were independently linked to worse cardiovascular health in cancer survivors (37). The stronger association of social isolation and loneliness with CVD mortality compared to cancer-specific mortality, possibly due to the predominant influence of tumor stage at diagnosis and cancer-specific management on oncologic outcomes, which are typically prioritized over cardiovascular care. Given that oncological problems are usually more clinically salient, cancer-specific mortality appears less susceptible to social connections than CVD mortality. Considering that social isolation and loneliness may exacerbate cardiovascular outcomes in cancer survivors, we recommend incorporating standardized social connection assessments into conventional cardiovascular risk stratification protocols to identify high-risk patients, enabling targeted interventions to mitigate long-term cardiovascular complications and mortality (38). Accordingly, interventions should prioritize individuals experiencing social isolation over those reporting loneliness alone, as the additional benefits of solely alleviating loneliness may be limited among those with high levels of social isolation. For individuals with elevated loneliness, greater attention should be directed toward their cardiovascular health.

The exploratory analyses revealed that living alone, as a key component of social isolation, was associated with a 29% to 84% increase in non-cancer mortality among survivors of breast, prostate, colorectal, and skin malignancies, as well as a 21% to 35% increase in all-cause mortality among survivors of prostate, colorectal, and skin malignancies. These findings aligned with previous research results observed in the general population (7, 39, 40). We found that among breast cancer survivors, weekly social activities were associated with lower all-cause and cancer mortality, and monthly family/friends visits were linked to lower all-cause and non-cancer mortality. Consistent with our results, several prospective cohorts also showed that social isolation was not associated with breast cancer recurrence or breast cancer-specific mortality, but was significantly associated with high all-cause mortality and mortality from other causes (41, 42). A large pooled cohort indicated that smaller social networks among breast cancer survivors were strongly associated with higher non-breast cancer mortality, but the relationship with breast cancer-specific mortality was more complex (43). Another population-based study of postmenopausal breast cancers showed an association between higher social support and lower all-cause mortality, regardless of cancer stages at diagnosis (44). Furthermore, our study found that loneliness was correlated with a 70% and 46% higher risk of non-cancer mortality in prostate and colorectal cancer survivors, respectively. Existing literature suggested that psychosocial interventions were effective in improving the quality of life for prostate cancer survivors and their partners (45, 46). Nonetheless, few studies have reported the impact of loneliness among prostate and colorectal cancer survivors.

Collectively, in the long-term follow-up of cancer survivors, comprehensive management of cardiovascular and other-cause risks should be strengthened alongside tumor recurrence surveillance, with particular attention to those living alone. Additionally, tailored psychosocial intervention strategies should be implemented for patients with different cancer types.

In terms of biological mechanisms, social isolation and loneliness can trigger activation of the hypothalamic-pituitary-adrenal (HPA) axis and sympathetic nervous system, leading to increased glucocorticoid secretion and treatment-related QT interval (47, 48). Glucocorticoids contribute to elevated systolic blood pressure and atherosclerotic progression through increasing vasoconstriction, decreasing endothelial nitric oxide production, and heightening oxidative stress (49). A cross-sectional study reported that living alone was associated with a dysregulated HPA axis and elevated C-reactive protein (CRP) levels, while loneliness was related to higher interleukin-6 levels (50). A study analyzing blood samples from older adults showed that higher levels of social participation and living with others were related to lower levels of inflammation (CRP, fibrinogen, and white blood cell count), while loneliness was negatively correlated with inflammatory modulation, such as higher levels of insulin-like growth factor-1 in those who rarely felt lonely (51). This indicates that social isolation and loneliness may influence health through different biological pathways that warrant further examination.

In summary, our findings support incorporating assessments of social connections (e.g., living arrangements, social contacts frequency) into routine care for cancer survivors, particularly for cardiovascular risk evaluation. Compared with loneliness, social isolation exhibits a stronger and more consistent association with mortality. Accordingly, intervention programs for cancer survivors may prioritize connecting socially isolated patients with peer-support groups, community resources, and structured social activities. For participants experiencing, cardiovascular monitoring and stress management strategies tend to yield greater benefits. Nevertheless, we emphasize that further interventional studies are warranted before integrating social connection measures into standardized risk prediction models, to confirm whether ameliorating social isolation or loneliness can improve the survival outcomes among cancer survivors.

## 5 Strengths and limitations

The current study has several notable strengths, including (1) a prospective study design that minimizes recall bias and strengthens temporal inference; (2) a substantial sample size of cancer survivors with data on social isolation and loneliness, providing adequate statistical power; (3) comprehensive covariate data enabling robust adjustment for potential confounders.

However, several limitations warrant consideration. First, the UK Biobank lacks longitudinal data on the duration and stability of social isolation and loneliness, precluding analysis of their cumulative effects as well as reverse-causality concerns arising in the later follow-up period. Second, the UK Biobank cohort is subject to healthy-volunteer bias and comprises predominantly White European participants, who tend to have better overall health and higher socioeconomic status, which limits the external validity of our findings. Third, the social isolation and loneliness indices derived from simplified questionnaire measures may not fully capture the complex phenomenon of social networks. Fourth, the UK Biobank did not provide detailed tumor stage, disease recurrence status and information on therapeutic drugs (such as anthracyclines with cardiotoxicity), which are critical prognostic factors. Fifth, over 50% of participants had ≥5-year survivorship at baseline, potentially biasing our results toward early-stage cancer survivors. Sixth, the observational design inherently limits causal inference.

## 6 Conclusions

This cohort study of UK Biobank cancer survivors suggested that social isolation was independently associated with increased risks of all-cause, cancer-specific, and CVD-related mortality, whereas loneliness was associated with elevated CVD mortality. Our findings support integrating social connections screening into cancer survivorship care, developing targeted psychosocial interventions, and implementing multidisciplinary approaches addressing both social and medical risk factors.

## Data Availability

The datasets presented in this article are not readily available because UK Biobank data are available in a public, open access repository. This research has been conducted using the UK Biobank Resource under Application Number 152047. The UK Biobank data are available on application to the UK Biobank (www.ukbiobank.ac.uk/). Requests to access the datasets should be directed to Yingying Liu, lyy202606@xjtu.edu.cn.
