# Supplementary material for: Social isolation and loneliness and mortality among cancer survivors: a prospective cohort study
Source: Front Oncol. 2026 Sep 9;16:1908858. doi: 10.3389/fonc.2026.1908858 (PMC13597224; doi:10.3389/fonc.2026.1908858)
Supplement: Supplementary file 1 [file SupplementaryFile1.docx]

Supplementary Material

**Table S1.** Evaluation of social isolation and loneliness in the UK Biobank

**Table S2.** Assessment of healthy diet score in the UK Biobank

**Table S3.** Definition and description of covariates in the UK Biobank

**Table S4.** The number and percentage of missing data for major covariate before multiple imputation

**Table S5.** Generalized variance inflation factors (GVIF) for all variables included in Cox models

**Table S6.** Associations of loneliness with risk of all-cause mortality and cause-specific mortality stratified by levels of social isolation status

**Table S7.** Associations of social isolation with risk of all-cause mortality and cause-specific mortality stratified by levels of loneliness status

**Table S8.** Interaction effects of social isolation and loneliness on risk of all-cause mortality and cause-specific mortality

**Table S9.** Association of components of social isolation and loneliness with risk of all-cause mortality and cause-specific mortality among cancer survivors

**Table S10.** Baseline prevalence of social isolation and loneliness across cancer subtypes

**Table S11.** Baseline prevalence of social isolation and loneliness across subgroups

**Table S12.** Models of association between social isolation and mortality among cancer survivors excluding missing data (n=20357)

**Table S13.** Models of association between loneliness and mortality among cancer survivors excluding missing data (n=20357)

**Table S14.** Models of association between social isolation and mortality among cancer survivors for 2 years or more (n=31880)

**Table S15.** Models of association between loneliness and mortality among cancer survivors for 2 years or more (n=31880)

**Table S16.** Fully adjusted model of association between social isolation and loneliness (as continuous variables) with mortality among cancer survivors (n=32524)

**Figure S1.** Flowchart of participants included in the analysis

**Figure S2.** Directed acyclic graph (DAG) indicating the associations among exposures, outcomes, and included covariates in the analyses

**Figure S3.** Models of association between loneliness and mortality among cancer survivors

**Figure S4.** Cumulative hazard of all-cause mortality, cancer mortality, CVD and other-cause mortality among cancer survivors by social isolation

**Figure S5.** Cumulative hazard of all-cause mortality, cancer mortality, CVD and other-cause mortality among cancer survivors by loneliness

**Figure S6.** Association between social isolation measures with all-cause, cancer and non-cancer mortality risk among breast cancer survivors

**Figure S7.** Association between social isolation measures with all-cause, cancer and non-cancer mortality risk among prostate cancer survivors

**Figure S8.** Association between social isolation measures with all-cause, cancer and non-cancer mortality risk among colorectal cancer survivors

**Figure S9.** Association between social isolation measures with all-cause, cancer and non-cancer mortality risk among skin malignancy survivors

**Figure S10.** Subgroup analyses of the association between loneliness and mortality risk among cancer survivors

**Figure S11.** Competing risk regression analysis of social isolation, loneliness, and mortality in cancer survivors

**Table S1.** **Evaluation of social isolation and loneliness in the UK Biobank**

| Exposures | UK Biobank field ID | Field names | ACE touchscreen questions | Points | Responses |
| --- | --- | --- | --- | --- | --- |
| Social isolation | 709 | Number in household | Including yourself, how many people are living together in your household? | 0 | Not living alone |
|  |  |  |  | 1 | Living alone |
|  | 1031 | Frequency of friend/family visits | How often do you visit friends or family or have them visit you? | 0 | Almost daily |
|  |  |  |  |  | 2-4 times a week |
|  |  |  |  |  | About once a week |
|  |  |  |  |  | About once a month |
|  |  |  |  | 1 | Once every few months |
|  |  |  |  |  | Never or almost never |
|  |  |  |  |  | No friends/family outside household |
|  | 6160 | Leisure/  social activities | Which of the following (sports club or gym, pub or social club, religious group, adult education class, other group activity) do you attend once a week or more often? | 0 | Sports club or gym |
|  |  |  |  |  | Pub or social club |
|  |  |  |  |  | Religious group |
|  |  |  |  |  | Adult education class |
|  |  |  |  |  | Other group activity |
|  |  |  |  | 1 | None of the above |
| Loneliness | 2020 | Loneliness, isolation | Do you often feel lonely? | 0 | No |
|  |  |  |  | 1 | Yes |
|  | 2110 | Able to confide | How often are you able to confide in someone close to you? | 0 | Almost daily |
|  |  |  |  |  | 2-4 times a week |
|  |  |  |  |  | About once a week |
|  |  |  |  |  | About once a month |
|  |  |  |  | 1 | Once every few months |
|  |  |  |  |  | Never or almost never |

**Table S2.** **Assessment of healthy diet score in the UK Biobank**

| Diet | UK Biobank field ID | Description | Healthy diet score |
| --- | --- | --- | --- |
| Vegetable | 1289 | Cooked vegetable intake | 1 for ≥4 tablespoons/day |
|  | 1299 | Salad/raw vegetable intake | 0 for <4 tablespoons/day |
| Fruit | 1309 | Fresh fruit intake | 1 for ≥3 pieces/day |
|  | 1319 | Dried fruit intake | 0 for <3 pieces/day |
| Fish | 1329 | Oily fish intake | 1 for ≥2 times/week |
|  | 1339 | Non-oily fish intake | 0 for <2 times/week |
| Processed meat | 1349 | Processed meat intake | 1 for <2 times/week |
| Unprocessed red meat | 1369 | Beef intake | 0 for ≥2 times/week |
|  | 1379 | Lamb/mutton intake | 1 for <2 times/week |
|  | 1389 | Pork intake | 0 for ≥2 times/week |

**Table S3. Definition and description of** **covariates in the UK Biobank**

| **Covariates** | **Date fields** | **Field names** | **Categorizations** | **Note** |
| --- | --- | --- | --- | --- |
| Age | 21022 | Age at recruitment | Continuous | Age at recruitment refers to the age of the participant on the day they attended an Initial Assessment Centre |
| Sex | 31 | Sex | 1) Female 2) Male | NHS derived and/or touchscreen questionnaire |
| Ethnic type | 21000 | Ethnic background | 1) White 2) Non-White | Except for Whites, all other races are classified as non-Whites. |
| Townsend deprivation index | 22189 | Townsend deprivation index at recruitment | Continuous | Townsend deprivation index (pre-UK Biobank enrollment), derived from census output areas, assigned based on participant postcode. |
| Employment | 6142 | Current employment status | 1) Paid employment/Self-employed 2) Retired or unemployed | In paid employment or self-employed coded as Paid employment/Self-employed, all other answers coded as Retired or unemployed |
| Education | 6138 | Qualifications | 1) College degree or above 2) High school or equivalent 3) Less than high school | Touchscreen question "Which of the following qualifications do you have?" |
| Diet | 1289; 1299; 1309; 1319; 1329; 1339; 1349; 1369;  1379;  1389 | Cooked vegetable intake; Salad/raw vegetable intake; Fresh fruit intake; Dried fruit intake; Oily fish intake; Non-oily fish intake; Processed meat intake; Beef intake; Lamb/mutton intake; Pork intake | Healthy  Unhealthy | Assessment of healthy diet score detailed in Table S2.  Healthy diet: ≥3 points;  Unhealthy diet: <3 points |
| Income | 738 | Average total household income before tax | 1) Less than £18,000 2) £18,000 to £30,999 3) £31,000 to £51,999 4) Greater than £52,000 | ACE touchscreen question "What is the average total income before tax received by your HOUSEHOLD?" |
| Smoking status | 20116 | Smoking status | 1) Current 2) Previous 3) Never | This field summarizes the current/past smoking status of the participant. |
| Alcohol intake | 1558 | Alcohol drinker status | 1) Daily or almost daily 2) 3-4 times a week 3) 1-2 times a week 4) Occasionally 5) Never | Touchscreen questionnaire: “About how often do you drink alcohol?" |
| Physical activity | 22032 | IPAQ activity group | 1) Low 2) Moderate 3) High | IPAQ groups |
| Body mass index (BMI) | 21001 | Body mass index | Continuous |  |
| Diabetes | 2443; 20002; 6177; 6153 | Diabetes diagnosed by doctor; Non-cancer illness code, self-reported; Medication for cholesterol, blood pressure or diabetes; Medication for cholesterol, blood pressure, diabetes, or take exogenous hormones | 1) Yes 2) No | Touchscreen questionnaire and verbal interview: "self-reported diabetes (diabetes, type 1 diabetes or type 2 diabetes) or insulin use." |
| Hypertension | 6150; 20002; 6177; 6153 | Vascular/heart problems diagnosed by doctor; Non-cancer illness code, self-reported; Medication for cholesterol, blood pressure or diabetes; Medication for cholesterol, blood pressure, diabetes, or take exogenous hormones | 1) Yes 2) No | Touchscreen questionnaire and verbal interview: "self-reported hypertension or anti-hypertensive medication use." |
| Hyperlipidemia | 20002; 6177; 6153 | Medication for cholesterol, blood pressure or diabetes; Medication for cholesterol, blood pressure, diabetes, or take exogenous hormones | 1) Yes 2) No | Touchscreen questionnaire and verbal interview: " high cholesterol or cholesterol lowering medication use." |
| Cardiovascular diseases (CVD) | 6150 | Vascular/heart problems diagnosed by doctor | 1) Yes 2) No | Touchscreen question: "Has a doctor ever told you that you have had any of the following conditions?" |
| Chronic lung disease | 6152 | Blood clot, DVT, bronchitis, emphysema, asthma, rhinitis, eczema, allergy diagnosed by doctor | 1) Yes 2) No | Touchscreen question "Has a doctor ever told you that you have had emphysema/chronic bronchitis?" |
| Self-reported health | 2178 | Overall health rating | 1) Excellent 2) Good 3) Fair 4) Poor   Continuous (ranged from 1 to 4) | Touchscreen question "In general how would you rate your overall health?" |
| Depressed mood | 2050 | Frequency of depressed mood in last 2 weeks | 1) Not at all 2) Several days 3) More than half the days 4) Nearly every day   Continuous (ranged from 1 to 4) | Touchscreen question "Over the past two weeks, how often have you felt down, depressed or hopeless?" |
| Unenthusiasm/disinterest | 2060 | Frequency of unenthusiasm / disinterest in last 2 weeks | 1) Not at all 2) Several days 3) More than half the days 4) Nearly every day   Continuous (ranged from 1 to 4) | Touchscreen question "Over the past two weeks, how often have you had little interest or pleasure in doing things?" |
| Tenseness/restlessness | 2070 | Frequency of tenseness / restlessness in last 2 weeks | 1) Not at all 2) Several days 3) More than half the days 4) Nearly every day   Continuous (ranged from 1 to 4) | Touchscreen question "Over the past two weeks, how often have you felt tense, fidgety or restless?" |
| Tiredness/lethargy | 2080 | Frequency of tiredness / lethargy in last 2 weeks | 1) Not at all 2) Several days 3) More than half the days 4) Nearly every day   Continuous (ranged from 1 to 4) | Touchscreen question "Over the past two weeks, how often have you felt tired or had little energy?" |

**Table S4. The number and percentage of missing data for major covariate before multiple imputation**

| Covariate | Missing data (n, %) |
| --- | --- |
| Sex | 0 (0) |
| Age | 0 (0) |
| Ethnicity | 77 (0.24) |
| Income | 560 (1.72) |
| Employment | 207 (0.64) |
| Education | 294 (0.9) |
| Townsend deprivation index | 36 (0.11) |
| Body mass index (BMI), kg/m^2^ | 151 (0.5) |
| Smoking status | 127 (0.46) |
| Alcohol intake | 14 (0.04) |
| Healthy diet | 5 (0.02) |
| Sleep duration, hours | 163 (0.50) |
| IPAQ | 2385 (7.33) |
| Hypertension | 0 (0) |
| Diabetes | 57 (0.18) |
| Hyperlipidemia | 0 (0) |
| Cardiovascular diseases (CVD) | 48 (0.15) |
| Chronic lung disease | 35 (0.11) |
| Self-rated health | 208 (0.64) |
| Depressed mood | 1220 (3.75) |
| Unenthusiasm/disinterest | 884 (2.72) |
| Tenseness/restlessness | 1086 (3.34) |
| Tiredness/lethargy | 871 (2.68) |

**Table S5. Generalized variance inflation factors** **(GVIF) for all variables included in Cox models**

| Variable | GVIF |
| --- | --- |
| Social isolation | 1.07 |
| Loneliness | 1.14 |
| Sex | 1.18 |
| Age | 1.61 |
| Ethnicity | 1.04 |
| Income | 1.59 |
| Employment | 1.60 |
| Education | 1.28 |
| Townsend index | 1.15 |
| Body mass index (BMI) | 1.16 |
| Smoking status | 1.09 |
| Alcohol intake | 1.13 |
| Healthy Diet | 1.07 |
| Sleep duration | 1.02 |
| IPAQ | 1.07 |
| Hypertension | 1.22 |
| Diabetes | 1.17 |
| Hyperlipidemia | 1.45 |
| Cardiovascular disease (CVD) | 1.21 |
| Chronic lung disease | 1.04 |
| Depressed mood | 1.95 |
| Unenthusiasm/disinterest | 1.79 |
| Tenseness/restlessness | 1.57 |
| Tiredness/lethargy | 1.46 |
| Self-rated health | 1.44 |
| Time since cancer diagnosis | 1.03 |

**Table S6.** **Associations of loneliness with risk of all-cause mortality and cause-specific mortality stratified by levels of social isolation status**

|  | Social isolation status | | | | | | | |
| --- | --- | --- | --- | --- | --- | --- | --- | --- |
|  | Low social isolation | | | | High social isolation | | | |
|  | N | Cases/Person-years | HR (95% CI) | *P* value | N | Cases/Person-years | HR (95% CI) | *P* value |
| All-cause mortality |  |  |  |  |  |  |  |  |
| Loneliness |  |  |  |  |  |  |  |  |
| Low | 21124 | 3583/301701 | 1.00 (Reference) |  | 1399 | 321/19254 | 1.00 (Reference) |  |
| High | 8426 | 1606/119709 | 0.98 (0.92–1.04) | 0.535 | 1575 | 435/21168 | 0.98 (0.84–1.15) | 0.831 |
| Cancer mortality |  |  |  |  |  |  |  |  |
| Loneliness |  |  |  |  |  |  |  |  |
| Low | 21124 | 2512/301701 | 1.00 (Reference) |  | 1399 | 207/19254 | 1.00 (Reference) |  |
| High | 8426 | 995/119709 | 0.89 (0.83–0.97) | 0.004 | 1575 | 251/21168 | 0.95 (0.78–1.15) | 0.585 |
| CVD mortality |  |  |  |  |  |  |  |  |
| Loneliness |  |  |  |  |  |  |  |  |
| Low | 21124 | 407/301701 | 1.00 (Reference) |  | 1399 | 39/19254 | 1.00 (Reference) |  |
| High | 8426 | 242/119709 | 1.29 (1.09–1.52) | 0.003 | 1575 | 74/21168 | 1.14 (0.75–1.72) | 0.551 |
| Other-cause mortality |  |  |  |  |  |  |  |  |
| Loneliness |  |  |  |  |  |  |  |  |
| Low | 21124 | 664/301701 | 1.00 (Reference) |  | 1399 | 75/19254 | 1.00 (Reference) |  |
| High | 8426 | 369/119709 | 1.12 (0.98–1.28) | 0.111 | 1575 | 110/21168 | 1.00 (0.73–1.37) | 0.979 |

Abbreviations: CI, confidence interval; CVD: cardiovascular disease; HR, hazard ratio.

Multivariable model: adjusted for age, sex, and ethnicity, income, employment, education, Townsend deprivation index, smoking status, alcohol intake, diet, sleep duration, IPAQ, BMI, hypertension, diabetes, hyperlipidemia, CVD, chronic lung disease, depressed mood, unenthusiasm/disinterest, tenseness/restlessness, tiredness/lethargy, self-rated health, and time since cancer diagnosis.

**Table S7.** **Associations of social isolation with risk of all-cause mortality and cause-specific mortality stratified by levels of loneliness status**

|  | Loneliness status | | | | | | | |
| --- | --- | --- | --- | --- | --- | --- | --- | --- |
|  | Low loneliness | | | | High loneliness | | | |
|  | N | Cases/Person-years | HR (95% CI) | *P* value | N | Cases/Person-years | HR (95% CI) | *P* value |
| All-cause mortality |  |  |  |  |  |  |  |  |
| Social isolation |  |  |  |  |  |  |  |  |
| Low | 21124 | 3583/301701 | 1.00 (Reference) |  | 8426 | 1606/119709 | 1.00 (Reference) |  |
| High | 1399 | 321/19254 | 1.22 (1.08–1.37) | 0.001 | 1575 | 435/21168 | 1.21 (1.08–1.35) | 0.001 |
| Cancer mortality |  |  |  |  |  |  |  |  |
| Social isolation |  |  |  |  |  |  |  |  |
| Low | 21124 | 2512/301701 | 1.00 (Reference) |  | 8426 | 995/119709 | 1.00 (Reference) |  |
| High | 1399 | 207/19254 | 1.14 (0.99–1.32) | 0.078 | 1575 | 251/21168 | 1.18 (1.02–1.36) | 0.028 |
| CVD mortality |  |  |  |  |  |  |  |  |
| Social isolation |  |  |  |  |  |  |  |  |
| Low | 21124 | 407/301701 | 1.00 (Reference) |  | 8426 | 242/119709 | 1.00 (Reference) |  |
| High | 1399 | 39/19254 | 1.34 (0.96–1.88) | 0.091 | 1575 | 74/21168 | 1.28 (0.98–1.68) | 0.075 |
| Other-cause mortality |  |  |  |  |  |  |  |  |
| Social isolation |  |  |  |  |  |  |  |  |
| Low | 21124 | 664/301701 | 1.00 (Reference) |  | 8426 | 369/119709 | 1.00 (Reference) |  |
| High | 1399 | 75/19254 | 1.49 (1.17–1.91) | 0.001 | 1575 | 110/21168 | 1.26 (1.01–1.57) | 0.041 |

Abbreviations: CI, confidence interval; CVD: cardiovascular disease; HR, hazard ratio.

Multivariable model: adjusted for age, sex, and ethnicity, income, employment, education, Townsend deprivation index, smoking status, alcohol intake, diet, sleep duration, IPAQ, BMI, hypertension, diabetes, hyperlipidemia, CVD, chronic lung disease, depressed mood, unenthusiasm/disinterest, tenseness/restlessness, tiredness/lethargy, self-rated health, and time since cancer diagnosis.

**Table S8.** **Interaction effects of social isolation and loneliness on risk of all-cause mortality and cause-specific mortality**

|  | Low loneliness  HR (95% CI) | High loneliness  HR (95% CI) | Multiplicative interaction  HR (95% CI) | Additive interaction | | |
| --- | --- | --- | --- | --- | --- | --- |
|  |  |  |  | RERI (95% CI) | AP (95% CI) | SI (95% CI) |
| All-cause mortality |  |  |  |  |  |  |
| Social isolation |  |  |  |  |  |  |
| Low | 1.00 (Reference) | 0.98 (0.92, 1.05) | 1 (0.86, 1.17) | 0 (-0.19, 0.18) | 0 (-0.16, 0.15) | 0.98 (0.39, 2.48) |
| High | 1.22 (1.08, 1.37) | 1.20 (1.08, 1.33) |  |  |  |  |
| Cancer mortality |  |  |  |  |  |  |
| Social isolation |  |  |  |  |  |  |
| Low | 1.00 (Reference) | 0.90 (0.83, 0.97) | 1.04 (0.85, 1.27) | 0.02 (-0.19, 0.24) | 0.02 (-0.18, 0.22) | 1.81 (0, 3.58) |
| High | 1.13 (0.98, 1.31) | 1.05 (0.92, 1.21) |  |  |  |  |
| CVD mortality |  |  |  |  |  |  |
| Social isolation |  |  |  |  |  |  |
| Low | 1.00 (Reference) | 1.26 (1.07–1.49) | 0.97 (0.63, 1.47) | 0.04 (-0.56, 0.64) | 0.02 (-0.34, 0.38) | 1.06 (0.41, 2.75) |
| High | 1.35 (0.97–1.88) | 1.65 (1.27–2.15) |  |  |  |  |
| Other-cause mortality |  |  |  |  |  |  |
| Social isolation |  |  |  |  |  |  |
| Low | 1.00 (Reference) | 1.14 (0.99–1.30) | 0.83 (0.6, 1.15) | -0.22 (-0.68, 0.24) | -0.15 (-0.49, 0.19) | 0.66 (0.28, 1.56) |
| High | 1.51 (1.18–1.92) | 1.43 (1.15–1.77) |  |  |  |  |

Abbreviations: AP, attributable proportion due to interaction; CI, confidence interval; CVD: cardiovascular disease; HR, hazard ratio; RERI, relative excess risk due to interaction; SI, the synergy index.

Multivariable model: adjusted for age, sex, and ethnicity, income, employment, education, Townsend deprivation index, smoking status, alcohol intake, diet, sleep duration, IPAQ, BMI, hypertension, diabetes, hyperlipidemia, CVD, chronic lung disease, depressed mood, unenthusiasm/disinterest, tenseness/restlessness, tiredness/lethargy, self-rated health, and time since cancer diagnosis.

**Table S9.** **Association of components of social isolation and loneliness with risk of all-cause mortality** **and cause-specific mortality among cancer survivors**

| Indicators | Index=0 | HR (95% CI) [Index=1] | | |
| --- | --- | --- | --- | --- |
|  |  | All-cause mortality | Cancer mortality | CVD mortality |
| Social isolation indicators | | | | |
| Live alone | 1.00 (Reference) | 1.21 (1.14–1.29) | 1.15 (1.15–1.24) | 1.38 (1.16–1.43) |
| Weekly social activities | 1.00 (Reference) | 1.06 (1.00–1.12) | 1.10 (1.03–1.18) | 1.00 (0.85–1.17) |
| Frequency of friend/family visits | 1.00 (Reference) | 1.12 (1.02–1.23) | 0.99 (0.87–1.12) | 1.30 (1.02–1.65) |
| Loneliness indicators | | | | |
| Often feeling lonely | 1.00 (Reference) | 0.97 (0.90–1.04) | 0.94 (0.86–1.03) | 1.19 (0.98–1.44) |
| Able to confide | 1.00 (Reference) | 1.00 (0.94–1.07) | 0.92 (0.85–1.00) | 1.22 (1.04–1.44) |

Abbreviations: CI, confidence interval; CVD: cardiovascular disease; HR, hazard ratio.

Multivariable model: adjusted for age, sex, and ethnicity, income, employment, education, Townsend deprivation index, smoking status, alcohol intake, diet, sleep duration, IPAQ, BMI, hypertension, diabetes, hyperlipidemia, CVD, chronic lung disease, depressed mood, unenthusiasm/disinterest, tenseness/restlessness, tiredness/lethargy, self-rated health, and time since cancer diagnosis.

**Table S10. Baseline prevalence of social isolation and loneliness across cancer subtypes**

|  | Social isolation | | Loneliness | |
| --- | --- | --- | --- | --- |
|  | Low  (n, %) | High  (n, %) | Low  (n, %) | High  (n, %) |
| Breast cancer  (n=8340) | 7587 (91.0) | 753 (9.0) | 5835 (70.0) | 2505 (30.0) |
| Prostate cancer  (n=2841) | 2595 (91.3) | 246 (8.7) | 1997 (70.3) | 844 (29.7) |
| Colorectal cancer  (n=2034) | 1821 (89.5) | 213 (10.5) | 1401 (68.9) | 633 (31.1) |
| Female reproductive malignancy  (n=2352) | 2072 (88.1) | 280 (11.9) | 1554 (66.1) | 798 (33.9) |
| Skin malignancy  (n=10741) | 9921 (92.4) | 820 (7.6) | 7538 (70.2) | 3203 (29.8) |
| Hematologic malignancy  (n=1850) | 1663 (89.9) | 187 (10.1) | 1279 (69.1) | 571 (30.9) |

**Table S11. Baseline prevalence of social isolation and loneliness across subgroups**

|  | Social isolation | | Loneliness | |
| --- | --- | --- | --- | --- |
|  | Low  (n, %) | High  (n, %) | Low  (n, %) | High  (n, %) |
| Age at baseline, years |  |  |  |  |
| <60 | 10660 (90.2) | 1157 (9.8) | 8139 (68.9) | 3678 (31.1) |
| ≥60 | 18890 (91.2) | 1817 (8.8) | 14384 (69.5) | 6323 (30.5) |
| Sex |  |  |  |  |
| Male | 11773 (91.0) | 1168 (9.0) | 8970 (69.3) | 3971 (30.7) |
| Female | 17777 (90.8) | 1806 (9.2) | 13553 (69.2) | 6030 (30.8) |
| Ethnicity |  |  |  |  |
| White | 28249 (91.1) | 2775 (8.9) | 21573 (69.5) | 9451 (30.5) |
| Non-white | 1301 (86.7) | 199 (13.3) | 950 (63.3) | 550 (36.7) |
| Townsend index (tertiles) |  |  |  |  |
| T1 | 9404 (86.0) | 1533 (14.0) | 7000 (64.0) | 3937 (36.0) |
| T2 | 10010 (92.5) | 814 (7.5) | 7662 (70.8) | 3162 (29.2) |
| T3 | 10136 (94.2) | 627 (5.8) | 7861 (73.0) | 2902 (27.0) |
| BMI, kg/m^2^ |  |  |  |  |
| <25 | 10022 (91.0) | 987 (9.0) | 7872 (71.5) | 3137 (28.5) |
| 25–30 | 12592 (91.6) | 1150 (8.4) | 9545 (69.5) | 4197 (30.5) |
| ≥30 | 6936 (89.2) | 837 (10.8) | 5106 (65.7) | 2667 (34.3) |
| Chronic diseases at baseline |  |  |  |  |
| No | 17193 (91.8) | 1541 (8.2) | 13299 (71.0) | 5435 (29.0) |
| Yes | 12357 (89.6) | 1433 (10.4) | 9224 (66.9) | 4566 (33.1) |
| Cancer diagnosis occurred |  |  |  |  |
| Over 5 years prior to the baseline | 16152 (90.9) | 1611 (9.1) | 12146 (68.4) | 5617 (31.6) |
| Within 5 years prior to the baseline | 13398 (90.8) | 1363 (9.2) | 10377 (70.3) | 4384 (29.7) |

Abbreviations: BMI, body mass index

**Table S12. Models of association between social isolation and mortality among cancer survivors excluding missing data (n=20357)**

|  | Social isolation | | |
| --- | --- | --- | --- |
|  | HR (95% CI) | | *P* value |
|  | Low | High |  |
| All-cause mortality |  |  |  |
| Cases/person-years (mortality rate [/1,000 PY]) | 3010/267259 (11.3) | 433/23824 (18.2) |  |
| Model 1 (Social isolation + Demographic factors ^a^) | 1.00 (Reference) | 1.67 (1.51–1.85) | <0.001 |
| Model 2 (Model 1 + Socioeconomic factors ^b^) | 1.00 (Reference) | 1.46 (1.31–1.62) | <0.001 |
| Model 3 (Model 2 + Lifestyle factors ^c^) | 1.00 (Reference) | 1.34 (1.21–1.49) | <0.001 |
| Model 4 (Model 3 + Health status ^d^) | 1.00 (Reference) | 1.28 (1.15–1.42) | <0.001 |
| Model 5 (Model 4 + Loneliness [Fully adjusted]) | 1.00 (Reference) | 1.28 (1.15–1.42) | <0.001 |
| Cancer mortality |  |  |  |
| Cases/person-years (mortality rate [/1,000 PY]) | 2087/267259 (7.8) | 267/23834 (11.2) |  |
| Model 1 (Social isolation + Demographic factors ^a^) | 1.00 (Reference) | 1.46 (1.28–1.66) | <0.001 |
| Model 2 (Model 1 + Socioeconomic factors ^b^) | 1.00 (Reference) | 1.33 (1.17–1.51) | <0.001 |
| Model 3 (Model 2 + Lifestyle factors ^c^) | 1.00 (Reference) | 1.23 (1.08–1.41) | 0.002 |
| Model 4 (Model 3 + Health status ^d^) | 1.00 (Reference) | 1.17 (1.03–1.34) | 0.019 |
| Model 5 (Model 4 + Loneliness [Fully adjusted]) | 1.00 (Reference) | 1.20 (1.05–1.37) | 0.008 |
| CVD mortality |  |  |  |
| Cases/person-years (mortality rate [/1,000 PY]) | 370/267259 (1.4) | 68/23834 (2.9) |  |
| Model 1 (Social isolation + Demographic factors ^a^) | 1.00 (Reference) | 2.26 (1.74–2.93) | <0.001 |
| Model 2 (Model 1 + Socioeconomic factors ^b^) | 1.00 (Reference) | 1.79 (1.37–2.33) | <0.001 |
| Model 3 (Model 2 + Lifestyle factors ^c^) | 1.00 (Reference) | 1.68 (1.28–2.20) | <0.001 |
| Model 4 (Model 3 + Health status ^d^) | 1.00 (Reference) | 1.57 (1.20–2.07) | 0.001 |
| Model 5 (Model 4 + Loneliness [Fully adjusted]) | 1.00 (Reference) | 1.49 (1.13–1.96) | 0.004 |
| Other-cause mortality |  |  |  |
| Cases/person-years (mortality rate [/1,000 PY]) | 553/267259 (2.1) | 98/23834 (4.1) |  |
| Model 1 (Social isolation + Demographic factors ^a^) | 1.00 (Reference) | 2.17 (1.75–2.69) | <0.001 |
| Model 2 (Model 1 + Socioeconomic factors ^b^) | 1.00 (Reference) | 1.74 (1.40–2.17) | <0.001 |
| Model 3 (Model 2 + Lifestyle factors ^c^) | 1.00 (Reference) | 1.56 (1.25–1.96) | <0.001 |
| Model 4 (Model 3 + Health status ^d^) | 1.00 (Reference) | 1.48 (1.19–1.86) | 0.001 |
| Model 5 (Model 4 + Loneliness [Fully adjusted]) | 1.00 (Reference) | 1.45 (1.15–1.82) | 0.001 |

^a^ Demographic factors: age, sex, ethnicity

^b^ Socioeconomic factors: income, employment, education, Townsend deprivation index

^c^ Lifestyle factors: smoking status, alcohol intake, health diet, sleep duration, IPAQ, BMI

^d^ Health status: hypertension, diabetes, hyperlipidemia, CVD, chronic lung disease, depressive symptoms (depressed mood, unenthusiasm/disinterest, tenseness/restlessness, tiredness/lethargy), self-rated health, time since cancer diagnosis

Abbreviations: HR, hazard ratio; CI, confidence interval; PY, person-years.

**Table S13. Models of association between loneliness and mortality among cancer survivors excluding missing data (n=20357)**

|  | Loneliness | | |
| --- | --- | --- | --- |
|  | HR (95% CI) | | *P* value |
|  | Low | High |  |
| All-cause mortality |  |  |  |
| Cases/person-years (mortality rate [/1,000 PY]) | 2313/207538 (11.1) | 1130/83546 (13.5) |  |
| Model 1 (Loneliness + Demographic factors ^a^) | 1.00 (Reference) | 1.21 (1.13–1.30) | <0.001 |
| Model 2 (Model 1 + Socioeconomic factors ^b^) | 1.00 (Reference) | 1.11 (1.03–1.19) | 0.007 |
| Model 3 (Model 2 + Lifestyle factors ^c^) | 1.00 (Reference) | 1.06 (0.98–1.14) | 0.138 |
| Model 4 (Model 3 + Health status ^d^) | 1.00 (Reference) | 1.00 (0.93–1.08) | 0.980 |
| Model 5 (Model 4 + Social isolation [Fully adjusted]) | 1.00 (Reference) | 0.98 (0.91–1.06) | 0.585 |
| Cancer mortality |  |  |  |
| Cases/person-years (mortality rate [/1,000 PY]) | 1655/207538 (8.0) | 699/83546 (8.4) |  |
| Model 1 (Loneliness + Demographic factors ^a^) | 1.00 (Reference) | 1.04 (0.96–1.14) | 0.345 |
| Model 2 (Model 1 + Socioeconomic factors ^b^) | 1.00 (Reference) | 0.93 (0.89–1.07) | 0.615 |
| Model 3 (Model 2 + Lifestyle factors ^c^) | 1.00 (Reference) | 0.94 (0.86–1.03) | 0.164 |
| Model 4 (Model 3 + Health status ^d^) | 1.00 (Reference) | 0.89 (0.81–0.98) | 0.014 |
| Model 5 (Model 4 + Social isolation [Fully adjusted]) | 1.00 (Reference) | 0.88 (0.80–0.96) | 0.006 |
| CVD mortality |  |  |  |
| Cases/person-years (mortality rate [/1,000 PY]) | 256/207538 (1.2) | 182/83546 (2.2) |  |
| Model 1 (Loneliness + Demographic factors ^a^) | 1.00 (Reference) | 1.77 (1.46–2.14) | <0.001 |
| Model 2 (Model 1 + Socioeconomic factors ^b^) | 1.00 (Reference) | 1.54 (1.27–1.87) | <0.001 |
| Model 3 (Model 2 + Lifestyle factors ^c^) | 1.00 (Reference) | 1.48 (1.22–1.80) | <0.001 |
| Model 4 (Model 3 + Health status ^d^) | 1.00 (Reference) | 1.39 (1.14–1.71) | 0.001 |
| Model 5 (Model 4 + Social isolation [Fully adjusted]) | 1.00 (Reference) | 1.34 (1.09–1.65) | 0.005 |
| Other-cause mortality |  |  |  |
| Cases/person-years (mortality rate [/1,000 PY]) | 402/207538 (1.9) | 249/83546 (3.0) |  |
| Model 1 (Loneliness + Demographic factors ^a^) | 1.00 (Reference) | 1.54 (1.32–1.81) | <0.001 |
| Model 2 (Model 1 + Socioeconomic factors ^b^) | 1.00 (Reference) | 1.34 (1.14–1.57) | <0.001 |
| Model 3 (Model 2 + Lifestyle factors ^c^) | 1.00 (Reference) | 1.26 (1.07–1.48) | 0.005 |
| Model 4 (Model 3 + Health status ^d^) | 1.00 (Reference) | 1.20 (1.01–1.42) | 0.037 |
| Model 5 (Model 4 + Social isolation [Fully adjusted]) | 1.00 (Reference) | 1.16 (0.98–1.38) | 0.091 |

^a^ Demographic factors: age, sex, ethnicity

^b^ Socioeconomic factors: income, employment, education, Townsend deprivation index

^c^ Lifestyle factors: smoking status, alcohol intake, health diet, sleep duration, IPAQ, BMI

^d^ Health status: hypertension, diabetes, hyperlipidemia, CVD, chronic lung disease, depressive symptoms (depressed mood, unenthusiasm/disinterest, tenseness/restlessness, tiredness/lethargy), self-rated health, time since cancer diagnosis

Abbreviations: HR, hazard ratio; CI, confidence interval; PY, person-years.

**Table S14.** **Models of association between social isolation and mortality among cancer survivors for 2 years or more (n=31880)**

|  | Social isolation | | |
| --- | --- | --- | --- |
|  | HR (95% CI) | | *P* value |
|  | Low | High |  |
| All-cause mortality |  |  |  |
| Cases/person-years (mortality rate [/1,000 PY]) | 4635/420802 (11.0) | 666/40324 (16.5) |  |
| Model 1 (Social isolation + Demographic factors ^a^) | 1.00 (Reference) | 1.56 (1.44–1.69) | <0.001 |
| Model 2 (Model 1 + Socioeconomic factors ^b^) | 1.00 (Reference) | 1.38 (1.27–1.50) | <0.001 |
| Model 3 (Model 2 + Lifestyle factors ^c^) | 1.00 (Reference) | 1.28 (1.18–1.40) | <0.001 |
| Model 4 (Model 3 + Health status ^d^) | 1.00 (Reference) | 1.23 (1.13–1.34) | <0.001 |
| Model 5 (Model 4 + Loneliness [Fully adjusted]) | 1.00 (Reference) | 1.23 (1.13–1.33) | <0.001 |
| Cancer mortality |  |  |  |
| Cases/person-years (mortality rate [/1,000 PY]) | 3036/420802 (7.2) | 389/40324 (9.6) |  |
| Model 1 (Social isolation + Demographic factors ^a^) | 1.00 (Reference) | 1.37 (1.23–1.52) | <0.001 |
| Model 2 (Model 1 + Socioeconomic factors ^b^) | 1.00 (Reference) | 1.27 (1.14–1.41) | <0.001 |
| Model 3 (Model 2 + Lifestyle factors ^c^) | 1.00 (Reference) | 1.19 (1.07–1.33) | 0.001 |
| Model 4 (Model 3 + Health status ^d^) | 1.00 (Reference) | 1.15 (1.03–1.29) | 0.010 |
| Model 5 (Model 4 + Loneliness [Fully adjusted]) | 1.00 (Reference) | 1.17 (1.05–1.31) | 0.005 |
| CVD mortality |  |  |  |
| Cases/person-years (mortality rate [/1,000 PY]) | 609/420802 (1.4) | 103/40324 (2.6) |  |
| Model 1 (Social isolation + Demographic factors ^a^) | 1.00 (Reference) | 1.91 (1.55–2.35) | <0.001 |
| Model 2 (Model 1 + Socioeconomic factors ^b^) | 1.00 (Reference) | 1.55 (1.25–1.92) | <0.001 |
| Model 3 (Model 2 + Lifestyle factors ^c^) | 1.00 (Reference) | 1.45 (1.17–1.80) | 0.001 |
| Model 4 (Model 3 + Health status ^d^) | 1.00 (Reference) | 1.37 (1.10–1.70) | 0.004 |
| Model 5 (Model 4 + Loneliness [Fully adjusted]) | 1.00 (Reference) | 1.31 (1.05–1.63) | 0.017 |
| Other-cause mortality |  |  |  |
| Cases/person-years (mortality rate [/1,000 PY]) | 990/420802 (2.4) | 173/40324 (4.3) |  |
| Model 1 (Social isolation + Demographic factors ^a^) | 1.00 (Reference) | 1.98 (1.68–2.32) | <0.001 |
| Model 2 (Model 1 + Socioeconomic factors ^b^) | 1.00 (Reference) | 1.61 (1.36–1.90) | <0.001 |
| Model 3 (Model 2 + Lifestyle factors ^c^) | 1.00 (Reference) | 1.46 (1.23–1.72) | <0.001 |
| Model 4 (Model 3 + Health status ^d^) | 1.00 (Reference) | 1.37 (1.16–1.62) | <0.001 |
| Model 5 (Model 4 + Loneliness [Fully adjusted]) | 1.00 (Reference) | 1.35 (1.14–1.59) | <0.001 |

^a^ Demographic factors: age, sex, ethnicity

^b^ Socioeconomic factors: income, employment, education, Townsend deprivation index

^c^ Lifestyle factors: smoking status, alcohol intake, health diet, sleep duration, IPAQ, BMI

^d^ Health status: hypertension, diabetes, hyperlipidemia, CVD, chronic lung disease, depressive symptoms (depressed mood, unenthusiasm/disinterest, tenseness/restlessness, tiredness/lethargy), self-rated health, time since cancer diagnosis

Abbreviations: HR, hazard ratio; CI, confidence interval; PY, person-years.

**Table S15.** **Models of association between loneliness and mortality among cancer survivors for 2 years or more (n=31880)**

|  | Loneliness | | |
| --- | --- | --- | --- |
|  | HR (95% CI) | | *P* value |
|  | Low | High |  |
| All-cause mortality |  |  |  |
| Cases/person-years (mortality rate [/1,000 PY]) | 3466/320481 (10.8) | 1835/140646 (13.0) |  |
| Model 1 (Loneliness + Demographic factors ^a^) | 1.00 (Reference) | 1.22 (1.15–1.29) | <0.001 |
| Model 2 (Model 1 + Socioeconomic factors ^b^) | 1.00 (Reference) | 1.12 (1.06–1.19) | 0.001 |
| Model 3 (Model 2 + Lifestyle factors ^c^) | 1.00 (Reference) | 1.07 (1.01–1.14) | 0.017 |
| Model 4 (Model 3 + Health status ^d^) | 1.00 (Reference) | 1.00 (0.94–1.06) | 0.958 |
| Model 5 (Model 4 + Social isolation [Fully adjusted]) | 1.00 (Reference) | 1.01 (0.95–1.07) | 0.780 |
| Cancer mortality |  |  |  |
| Cases/person-years (mortality rate [/1,000 PY]) | 2343/320481 (7.3) | 1082/140646 (7.7) |  |
| Model 1 (Loneliness + Demographic factors ^a^) | 1.00 (Reference) | 1.06 (0.98–1.14) | 0.129 |
| Model 2 (Model 1 + Socioeconomic factors ^b^) | 1.00 (Reference) | 1.00 (0.93–1.08) | 0.969 |
| Model 3 (Model 2 + Lifestyle factors ^c^) | 1.00 (Reference) | 0.96 (0.90–1.04) | 0.315 |
| Model 4 (Model 3 + Health status ^d^) | 1.00 (Reference) | 0.94 (0.87–1.01) | 0.094 |
| Model 5 (Model 4 + Social isolation [Fully adjusted]) | 1.00 (Reference) | 0.93 (0.86–1.00) | 0.049 |
| CVD mortality |  |  |  |
| Cases/person-years (mortality rate [/1,000 PY]) | 416/320481 (1.3) | 296/140646 (2.1) |  |
| Model 1 (Loneliness + Demographic factors ^a^) | 1.00 (Reference) | 1.66 (1.43–1.92) | <0.001 |
| Model 2 (Model 1 + Socioeconomic factors ^b^) | 1.00 (Reference) | 1.47 (1.26–1.71) | <0.001 |
| Model 3 (Model 2 + Lifestyle factors ^c^) | 1.00 (Reference) | 1.39 (1.19–1.62) | <0.001 |
| Model 4 (Model 3 + Health status ^d^) | 1.00 (Reference) | 1.32 (1.12–1.54) | 0.001 |
| Model 5 (Model 4 + Social isolation [Fully adjusted]) | 1.00 (Reference) | 1.28 (1.09–1.51) | 0.004 |
| Other-cause mortality |  |  |  |
| Cases/person-years (mortality rate [/1,000 PY]) | 707/320481 (2.2) | 457/140646 (3.2) |  |
| Model 1 (Loneliness + Demographic factors ^a^) | 1.00 (Reference) | 1.50 (1.33–1.69) | <0.001 |
| Model 2 (Model 1 + Socioeconomic factors ^b^) | 1.00 (Reference) | 1.32 (1.17–1.49) | <0.001 |
| Model 3 (Model 2 + Lifestyle factors ^c^) | 1.00 (Reference) | 1.24 (1.10–1.39) | 0.001 |
| Model 4 (Model 3 + Health status ^d^) | 1.00 (Reference) | 1.14 (1.01–1.30) | 0.039 |
| Model 5 (Model 4 + Social isolation [Fully adjusted]) | 1.00 (Reference) | 1.11 (0.98–1.26) | 0.106 |

^a^ Demographic factors: age, sex, ethnicity

^b^ Socioeconomic factors: income, employment, education, Townsend deprivation index

^c^ Lifestyle factors: smoking status, alcohol intake, health diet, sleep duration, IPAQ, BMI

^d^ Health status: hypertension, diabetes, hyperlipidemia, CVD, chronic lung disease, depressive symptoms (depressed mood, unenthusiasm/disinterest, tenseness/restlessness, tiredness/lethargy), self-rated health, time since cancer diagnosis

Abbreviations: HR, hazard ratio; CI, confidence interval; PY, person-years.

**Table S16.** **Fully adjusted model of association between social isolation and loneliness (as continuous variables) with mortality among cancer survivors (n=32524)**

|  | All-cause mortality | | Cancer mortality | | CVD mortality | | Other-cause mortality | |
| --- | --- | --- | --- | --- | --- | --- | --- | --- |
|  | HR (95% CI) | *P* value | HR (95% CI) | *P* value | HR (95% CI) | *P* value | HR (95% CI) | *P* value |
| Social isolation | 1.12 (1.08–1.17) | <0.001 | 1.11 (1.06–1.16) | <0.001 | 1.15 (1.03–1.28) | 0.010 | 1.17 (1.08–1.27) | <0.001 |
| Loneliness | 0.97 (0.93–1.10) | 0.166 | 0.92 (0.87–0.97) | 0.004 | 1.15 (1.02–1.30) | 0.022 | 1.02 (0.92–1.12) | 0.725 |

Multivariable model: adjusted for age, sex, and ethnicity, income, employment, education, Townsend deprivation index, smoking status, alcohol intake, diet, sleep duration, IPAQ, BMI, hypertension, diabetes, hyperlipidemia, CVD, chronic lung disease, depressed mood, unenthusiasm/disinterest, tenseness/restlessness, tiredness/lethargy, self-rated health, time since cancer diagnosis, social isolation, and loneliness.

Abbreviations: HR, hazard ratio; CI, confidence interval

**Figure S1.** **Flowchart of participants included in the analysis**

**Figure S2.** **Directed acyclic graph (DAG) indicating the associations among exposures, outcomes, and included covariates in the analyses**

Demographic characteristics (age, sex, ethnicity) may act as confounders; socioeconomic status (income, employment, education, Townsend deprivation index), lifestyle factors (smoking status, alcohol intake, health diet, sleep duration, physical activity, body mass index), mental health (depressed mood, unenthusiasm/disinterest, tenseness/restlessness, tiredness/lethargy), and health condition (hypertension, diabetes, hyperlipidemia, cardiovascular diseases, chronic lung disease, self-rated health, time since cancer diagnosis) may act as confounders or mediators or both in the association between social isolation/loneliness and mortality risk.

DAG was drawn using <http://www.dagitty.net/>

**Figure S3.** **Models of association between loneliness and mortality among cancer survivors**

^a^ Demographic factors: age, sex, ethnicity

^b^ Socioeconomic factors: income, employment, education, Townsend deprivation index

^c^ Lifestyle factors: smoking status, alcohol intake, health diet, sleep duration, IPAQ, BMI

^d^ Health status: hypertension, diabetes, hyperlipidemia, CVD, chronic lung disease, depressive symptoms (depressed mood, unenthusiasm/disinterest, tenseness/restlessness, tiredness/lethargy), self-rated health, time since cancer diagnosis

Abbreviations: HR, hazard ratio; CI, confidence interval; PY, person-years.

**Figure S4.** **Cumulative hazard of all-cause mortality, cancer mortality, CVD and other-cause mortality among cancer survivors by social isolation**

Cumulative hazard curves of all-cause mortality (A), cancer (B), CVD (C), and other-cause (D) mortality in cancer survivors by social isolation.

Abbreviations: CVD, cardiovascular disease

**Figure S5.** **Cumulative hazard of all-cause mortality, cancer mortality, CVD and other-cause mortality among cancer survivors by loneliness**

Cumulative hazard curves of all-cause mortality (A), cancer (B), CVD (C), and other-cause (D) mortality in cancer survivors by loneliness.

Abbreviations: CVD, cardiovascular disease

**Figure S6.** **Association between social isolation measures with all-cause, cancer and non-cancer mortality risk among breast cancer survivors**

Abbreviations: HR, hazard ratio; CI, confidence interval; ref, reference.

Multivariable model: adjusted for age, sex, and ethnicity, income, employment, education, Townsend deprivation index, smoking status, alcohol intake, diet, sleep duration, IPAQ, BMI, hypertension, diabetes, hyperlipidemia, CVD, chronic lung disease, depressed mood, unenthusiasm/disinterest, tenseness/restlessness, tiredness/lethargy, self-rated health, time since cancer diagnosis, and loneliness.

**Figure S7.** **Association between social isolation measures with all-cause, cancer and non-cancer mortality risk among prostate cancer survivors**

Abbreviations: HR, hazard ratio; CI, confidence interval; ref, reference.

Multivariable model: adjusted for age, sex, and ethnicity, income, employment, education, Townsend deprivation index, smoking status, alcohol intake, diet, sleep duration, IPAQ, BMI, hypertension, diabetes, hyperlipidemia, CVD, chronic lung disease, depressed mood, unenthusiasm/disinterest, tenseness/restlessness, tiredness/lethargy, self-rated health, time since cancer diagnosis, and loneliness.

**Figure S8.** **Association between social isolation measures with all-cause, cancer and non-cancer mortality risk among colorectal cancer survivors**

Abbreviations: HR, hazard ratio; CI, confidence interval; ref, reference.

Multivariable model: adjusted for age, sex, and ethnicity, income, employment, education, Townsend deprivation index, smoking status, alcohol intake, diet, sleep duration, IPAQ, BMI, hypertension, diabetes, hyperlipidemia, CVD, chronic lung disease, depressed mood, unenthusiasm/disinterest, tenseness/restlessness, tiredness/lethargy, self-rated health, time since cancer diagnosis, and loneliness.

**Figure S9.** **Association between social isolation measures with all-cause, cancer and non-cancer mortality risk among skin malignancy survivors**

Abbreviations: HR, hazard ratio; CI, confidence interval; ref, reference.

Multivariable model: adjusted for age, sex, and ethnicity, income, employment, education, Townsend deprivation index, smoking status, alcohol intake, diet, sleep duration, IPAQ, BMI, hypertension, diabetes, hyperlipidemia, CVD, chronic lung disease, depressed mood, unenthusiasm/disinterest, tenseness/restlessness, tiredness/lethargy, self-rated health, time since cancer diagnosis, and loneliness.

**Figure S10.****Subgroup analyses of the association between loneliness and mortality risk among cancer survivors**

The dots represent the HRs for high versus low loneliness within the subgroups, and the horizontal lines indicate the 95% CIs.

Abbreviations: BMI, body mass index; CVD, cardiovascular disease; HR, hazard ratio; CI, confidence interval; ref, reference; PY, person-years.

**Figure S11. Competing risk regression analysis of social isolation, loneliness, and mortality in cancer survivors**
